# Histamine-Mediated Syndrome (HMS): Beyond Allergy and Therapeutic Potential of Immunoglobulin/Histamine Complex (IHC)

**DOI:** 10.3390/ijms27104494

**Published:** 2026-05-17

**Authors:** Hyuk Soon Kim, Geunwoong Noh

**Affiliations:** 1 Department of Biomedical Sciences, College of Natural Science, Dong-A University, Busan 49315, Republic of Korea; hskimxo@dau.ac.kr; 2 Department of Health Sciences, The Graduate School of Dong-A University, Busan 49315, Republic of Korea; 3 Department of Allergy and Clinical Immunology, Cheju Halla General Hospital, Jeju 63127, Republic of Korea

**Keywords:** histamine-mediated syndrome, histamine, mast cell, allergic disease, immunoglobulin/histamine complex

## Abstract

Histamine, present in almost all tissues, exerts diverse biologic actions through four receptors expressed across multiple organs and cell types. Histamine concentrations are elevated in various conditions. A wide spectrum of clinical manifestations where histamine levels increase has been described. When plasma histamine concentrations are increased, multiple symptoms may occur via histamine receptor-mediated signaling. These clinical situations are defined as histamine-mediated syndrome (HMS). Recent reports describe the use of an immunoglobulin/histamine complex (IHC) to treat conditions beyond classical allergic diseases, such as allergic rhinitis (AR) and chronic urticaria (CU). Baseline plasma histamine levels in AR and CU can be higher than expected. This suggests histamine-driven comorbid manifestations in tissues and organs expressing histamine receptors. Accordingly, HMS may be more common in routine practice than is generally appreciated. IHC may represent a rational therapeutic option for HMS. In this review, we define HMS and integrate the available evidence to propose a systematic clinical framework.

## 1. Introduction

Histamine, a biogenic amine formed by the decarboxylation of histidine, is present in almost all human tissues [1]. After early observations linking pollen exposure to hay fever, and the subsequent identification of histamine as a key mediator of allergic symptoms [2], histamine has been recognized as a major mediator of allergic diseases and anaphylaxis [3]. Meanwhile, accumulating evidence highlights the additional pathophysiologic roles of histamine beyond classical allergy.

Histamine has long been known as a mediator that plays a central role in allergies. However, the four histamine receptors are distributed across diverse cell types, including neurons, throughout the body [1]. Additional roles of histamine have been reported, and revisiting histamine biology from a broader clinical perspective is therefore timely.

Despite these broader roles, histamine is still primarily regarded in routine practice as a mediator of Immunoglobulin (Ig)E-mediated allergic diseases, such as allergic rhinitis (AR) and chronic urticaria (CU), as well as a regulator of gastric acid secretion. However, accumulating clinical observations suggest that histamine-associated manifestations can extend beyond classical allergies and involve multiple organ systems. Several reports have described improvements in both allergic and extra-allergic manifestations during immunoglobulin/histamine complex (IHC) therapy, raising the possibility of a broader histamine-driven clinical phenotype. In this context, histamine-related syndromic concepts have been proposed [4]. Here, we introduce the histamine-mediated syndrome (HMS) as a clinically oriented framework in which histamine-associated manifestations involve at least two organ systems, either concurrently or episodically, and may occur alongside or independent of classical allergic diseases. IHC is a histamine-fixed immunoglobulin preparation that has been reported to inhibit antigen-induced histamine release from human peripheral blood basophils and rat peritoneal mast cells [5], and it has been used as an anti-allergic therapy in clinical practice [6]. Proposed mechanisms include histamine sequestration or neutralization and, in some reports, the induction of antihistamine antibodies [7].

To support the HMS framework, we summarize key aspects of histamine biology, including synthesis, metabolism, cellular sources, and the distribution of histamine receptors, with an emphasis on their pathophysiological relevance to multi-organ symptom patterns and the clinical rationale for IHC.

## 2. Histamine Biology

Histamine biology was the beginning of understanding the HMS. Histamine was first described by Fühler in 1912 [8]. The name reflects its derivation from histidine metabolism, first described by Albrecht Kossel in 1889. Histamine was prepared synthetically by Windaus and Vogt in 1907 and was isolated from ergot by Sir Henry Dale and colleagues in 1910 [9]. It stimulated smooth muscles from the gut and the respiratory tract, which caused vasodepression, cardiac contractility, and, when injected into animals, induced a shock-like syndrome.

Popielski in 1920 demonstrated that histamine stimulated gastric acid secretion [10], and in 1927, Best, Dale, Dudley, and Thorpe proved that histamine was a natural constituent of the body by isolating this amine from the liver and the lung [11]. When histamine was demonstrated by Lewis and Grant, it was termed ‘H substance’ and was shown to be released from the skin by antigen–antibody interactions, the substance being found to be 2–[4–imidazolyl]–ethylamine, also known as β–aminoethylimidazole [12]. Three years later, Best and colleagues detected histamine in human tissue [11]. In 1932, histamine was identified as a mediator of anaphylactic reaction [13] and found to play a central role in the pathogenesis of several allergic diseases, such as allergic asthma, AR, and atopic dermatitis (AD), through differential regulation of T–helper lymphocytes [14,15]. However, histamine has also been found to mediate numerous biological processes, both physiologic and pathologic, some of which are related to allergic diseases [8]. The possibility was even raised that most of all human tissues contain histamine (ranging (<1 to >100) mg per gram of tissue) [16].

### 2.1. Histamine Synthesis

Histamine is produced in mammals and some bacteria, including gram–positive bacteria, such as Lactobacillus 30a [17] and Clostridium perfringens, and gram–negative bacteria [17,18]. The histamine production capacity of several cell types, including mast cells (MCs), basophils, certain neurons, and monocytes, raises the possibility that all human tissues contain histamine [8]. From this aspect, insight into histamine indicates that histamine appears to be a preparation for immunological defense. Since 1953, MCs and basophils have been reported to be the main sources of histamine production and release [19], which makes a pool of histamine that plays a central role in allergic reactions. Several cell types, such as lymphocytes (0.05 pg per cell), monocytes (0.05 pg per cell), macrophages, fibroblasts and platelets, parietal cells, and neurons of the central nervous system (CNS), also contain histamine [20,21]. However, unlike MCs and basophils, these cells do not store histamine and release it on production [22,23]. It plays a role in their cross-talk with surrounding cells and exerts biological functions in target cells.

MCs and basophils are the main cellular source of histamine. MCs have a relatively higher portion of histamine concentration, compared to basophils at ((0.1–0.2) vs. 0.01) pmol per cell, respectively [14]. Higher histamine levels are reported in MCs that are found in abundance in the lungs, skin, and gastrointestinal tract. Dermatologic findings provide a significant correlation between MC density and histamine levels in skin layers, supporting the notion that MCs are the main source of histamine in tissues [19,22]. However, a significant portion of the histamine in the human body comes from the intake of food and from gut microbiota [24]. This histamine is catabolized by diamine oxidase, which is continuously produced in the mucosa of the small intestine [25]. These cellular sources and tissue reservoirs explain why histamine-driven manifestations in HMS can occur in multiple organs.

### 2.2. Histamine Metabolism

The metabolism of histamine mainly occurs by two enzymatic pathways, through the activities of histamine *N*–methyltransferase and diamine oxidase or histaminase [26,27]. Most histamine is metabolized through the histamine *N*–methyltransferase pathway, which is in the CNS, intestinal smooth muscle, mucosa of the small intestine, liver, and kidneys [28]. The remaining histamine is metabolized through the diamine oxidase pathway, located in the small intestine mucosa, liver, kidneys, eosinophils, placenta, and skin. The major metabolic product is *N*–methylimidazole acetic acid. Accordingly, impaired histamine degradation may increase the systemic histamine burden, contributing to the persistence or recurrence of HMS-related symptom clusters.

### 2.3. Histamine Receptors

Histamine exerts diverse physiological and pathological effects by binding four distinct G protein–coupled receptors that are widely expressed across cells and tissues [29].

Histamine receptor 1 (H1R) is a key mediator of type 1 hypersensitivity reaction, and is expressed in multiple cell types, including MCs [15]. Histamine receptor 2 (H2R) is prominent in the gastrointestinal tract and regulates gastric acid secretion [30]. Presynaptic autoreceptor histamine receptor 3 (H3R) is abundantly expressed in the nervous system and contributes to the negative feedback control of histamine release [31]. Histamine receptor 4 (H4R) is expressed in the skin and in immune cells, particularly MCs and basophils, and participates in immunomodulatory signaling [32,33].

#### 2.3.1. H1 Receptor (H1R)

H1R is located in blood vessels and sensory nerves [10]. The most important activities of H1R are to increase vascular permeability, stimulate the sensory nerves of airways, and promote the chemotaxis of eosinophils. Therefore, it can cause sneezing, nasal congestion, and rhinorrhea in rhinitis. H1R is the primary receptor responsible for the symptoms of rhinitis. H1R antihistamines act as inverse agonists, causing a shift in equilibrium of the H1R to the inactivated state when bound to the H1R antihistamine [34].

#### 2.3.2. H2 Receptor (H2R)

H2R has always been regarded as a receptor for gastric acid, which is likely its major activity. However, it is located on the vascular bed, and it is known that to block all the systemic activities of histamine, a combination of both H1R and H2R antihistamines is necessary in rhinitis [35,36,37].

A combination of H1R and H2R activation can contribute to nasal airway swelling and rhinorrhea. The H1R and H2R antihistamines were found to significantly reduce rhinorrhea and provide a diminished reduction in nasal blood flow due to allergens. H1R antihistamine significantly reduced sneezing, whereas H2R antihistamine alone did not [38].

#### 2.3.3. H3 Receptor (H3R)

Regarding rhinitis, the importance of the H3 receptor is likely because it is expressed on presynaptic nerves in the peripheral sympathetic adrenergic system, and also on the nasal submucosal glands. Stimulation of the H3R receptor causes suppression of norepinephrine release at presynaptic nerve endings and stimulates nasal submucosal gland secretion [39]. In a cat model of rhinitis, it was found that H3R blockade enhanced the decongestant effect of H1R antihistamine [40].

#### 2.3.4. H4 Receptor (H4R)

The H4R can be found on eosinophils, mast cells, basophils, neutrophils, and nerves in the nasal turbinates. It is also seen in the lungs, colon, epicanthus, bone marrow, spleen, and liver [41]. Activation of H4R can enhance the chemotaxis and chemokinesis of mast cells and eosinophils [42]. H4R stimulation could theoretically enhance the inflammatory activity seen in AR.

The widespread distribution of H1R–H4R across various tissues provides a pathophysiological explanation for the multi-organ phenotype characteristic of HMS.

### 2.4. Histamine Level of Plasma and Tissue

Histamine regulates a plethora of pathophysiological and physiological processes, such as the secretion of gastric acid, inflammation, and the regulation of vasodilatation and bronchoconstriction [43,44]. In addition, it can also serve as a neurotransmitter [45].

Histamine was significantly produced, and its levels increased in allergic reactions, including anaphylaxis [13]. Research has found the baseline plasma level of histamine in normal subjects to be (0.3 to 0.4) ng/mL [46], while in anaphylaxis, the plasma level of histamine was reported to increase to 8.24 ng/mL [16]. Although the total whole blood histamine levels in humans ranges (50–60) ng/mL, most of this is stored in the granules of circulating basophils [8]. Histamine is rapidly degraded by enzymatic activity in plasma, resulting in lower measured levels than in whole blood, which contains histamine-rich basophils [11,47,48]. Compared to basophils, MCs have a relatively higher portion of this concentration of approximately ten–fold [14]. Histamine is a mediator for allergic reactions, which appear to be one of the defense mechanisms of the human body. In this aspect, ample histamine seems to exist in tissue and blood as preparation for body defense.

#### 2.4.1. Necessity of Standardization of Histamine Level: Histamine Index

Interpretation, comparison, and integration of the clinical significance of plasma histamine concentration is somewhat difficult due to differences in research findings, such as detection methods and their sensitivities. Therefore, standardization seems to be necessary to integrate the biological meaning according to the histamine concentration of the overall data from other laboratories. The concept of a histamine index (HI) may be necessary. Although plasma histamine concentration has been measured as a unit of ng/mL, HI is defined as the ratio of the histamine concentration of specific conditions to that of the baseline. For example, the baseline plasma concentration is (0.3–0.4). The HI of the baseline becomes one. The HI of plasma abruptly increases to about 21 (8.24/0.4), but after one day in anaphylaxis, it also rapidly decreases to about 0.5 (0.19/0.4).

The histamine concentration of whole blood is (50–60) ng/mL, while the HI of whole blood is about (125–150) (50/0.4 to 60/0.4). All human tissues contain histamine (HI ranging from <2.5 (1/0.4) to >2500 (100/0.4) mg per gram of tissue). Namely, histamine is always available in human tissues, and in particular, the availability of histamine in whole blood greatly exceeds the plasma level of histamine in anaphylaxis.

#### 2.4.2. Histamine in Anaphylaxis

**Figure 1:**
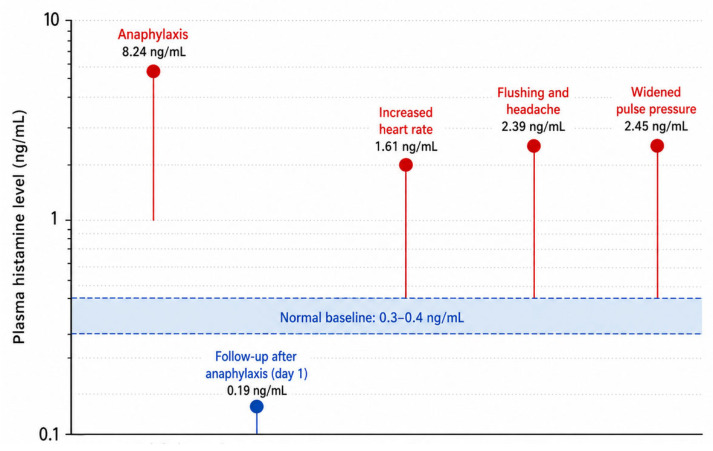
Plasma histamine levels and symptom thresholds. Normal baseline plasma histamine is reported to be approximately (0.3 to 0.4) ng/mL [46]. In anaphylaxis, plasma histamine has been reported to rise to about 8.2 ng/mL, and on the next day, decrease to about 0.19 ng/mL. Symptoms occurred at defined plasma histamine thresholds in the infusion study. Flushing and headache occurred at 2.39 ng/mL, increased heart rate at 1.61 ng/mL, and widened pulse pressure at 2.45 ng/mL [49].

The baseline plasma histamine level ranges from 0.3 to 0.4 ng/mL [46], while levels can rise to 8.24 ng/mL during anaphylaxis [13] (Figure 1). This rapid increase aligns with the acute release of mediators following mast cell and basophil degranulation in response to anaphylactic triggers. Plasma histamine levels subsequently decrease significantly within 24 h, whereas intracellular histamine levels exhibit an inverse pattern, reflecting the depletion and subsequent replenishment of cellular histamine stores. Consequently, the median intracellular histamine level in patients with anaphylaxis at admission was significantly lower than that in healthy controls (16.4 vs. 62.3 ng/mL) [16].

In addition to serving as a biomarker of acute activation, histamine plays a crucial role in the key clinical features of anaphylaxis through receptor-mediated effects. It promotes vasodilation and increases vascular permeability via H1R and H2R signaling, contributing to hypotension and edema. Histamine also induces bronchoconstriction and pruritus, primarily through H1R-mediated pathways. Together, these effects help elucidate the rapid onset of multi-organ symptoms during anaphylaxis.

## 3. Histamine–Mediated Syndrome (HMS)

HMS refers to a clinical condition in which histamine-related manifestations affect at least two organ systems, either concurrently or episodically. Within this framework, organ-specific symptom patterns can be analyzed in relation to histamine receptor distribution and histamine burden. We summarize representative conditions associated with increased plasma histamine in Table 1 and organ-specific manifestations in Table 2.

### 3.1. Definition of HMS

A wide range of clinical situations has been described in conditions in which histamine levels increase (Table 1). For example, mast cell activation syndrome (MCAS) can affect two or more organ systems and present with urticaria, angioedema, flushing, nausea, vomiting, diarrhea, abdominal cramping, hypotensive syncope, tachycardia, wheezing, conjunctival injection, pruritus, and nasal stuffiness [44]. Building on this multi-organ concept, HMS is defined as a clinical entity in which histamine-associated manifestations involve at least two organ systems, either concurrently or episodically.

Historically, plasma histamine was considered undetectable in healthy individuals, at less than 1 ng/mL, but measurable in patients with mastocytosis or chronic myelocytic leukemia (CML) [47]. Later studies established a baseline plasma histamine level of (0.3 to 0.4) ng/mL [46]. MCs and basophils are the major sources of histamine production and release, and form a pool linked to allergic reactions. In allergy-focused practice, baseline plasma histamine is often interpreted as reflecting prior or ongoing allergic provocation. However, because histamine exerts multiple biological functions through four receptors across diverse tissues and organs, elevations in plasma histamine in chronic conditions warrant broader clinical interpretation.

Anaphylaxis is an acute condition in which plasma histamine abruptly increases. Chronic disorders associated with increased plasma histamine include mastocytosis and CML. In these diseases, elevated plasma histamine has been associated with symptoms like hyperchlorhydria, transient facial flushing, pruritus, and urticaria [47] (Table 2).

MCs and basophils can rapidly release histamine and achieve high local concentrations in surrounding tissues [50]. Enterochromaffin-like cells produce local histamine that acts through H2R on parietal cells to induce gastric acid secretion [51]. Histaminergic neurons in the tuberomammillary nucleus produce histamine that signals via H1R and contribute to wake promotion and rapid eye movement sleep regulation [52,53]. These observations support a syndromic concept in which clusters of symptoms across organs may be driven by histamine signaling and may occur alongside, or independent of, classical allergic disease.

Because circulating histamine is metabolized rapidly, in the order of minutes [54], a single plasma measurement may be inadequate for diagnosis. Nevertheless, plasma histamine is useful for conceptualizing HMS and for linking histamine biology to multi-organ clinical manifestations.

### 3.2. Conditions Associated with Increased Plasma Histamine

Conditions that may increase plasma histamine include release from basophils, tissue injury, venous stasis, hemolysis, or clotting during blood collection and sample preparation, improper centrifugation or storage, and the ingestion of histamine-rich foods [54]. For clinical framing, these settings can be grouped as increased histamine production, impaired metabolism, increased sources of production, and increased dietary intake (Table 1).

Anaphylaxis is a representative condition in which plasma histamine increases abruptly [16], and can involve multiple organ systems, with patterns varying across patients, and across episodes in the same patient. Skin involvement is reported in (80 to 90) % of episodes, respiratory involvement in up to 70%, gastrointestinal involvement in up to 45%, cardiovascular involvement in up to 45%, and central nervous system involvement in up to 15% [55].

In drug allergy, resting baseline plasma histamine levels are often not markedly elevated. For example, in aspirin-induced urticaria, the resting plasma histamine level was (0.95 ± 0.25) ng/mL, showing only a mild increase, despite the provocation severity in acute situations of some patients [56].

Histamine is also present at high concentrations in AD [57]. Elevated histamine has been detected in both lesional and non-lesional skin in AD, supporting a role in pruritus pathogenesis [58]. However, tissue histamine levels and circulating histamine levels may reflect distinct aspects of HMS.

**Figure 2:**
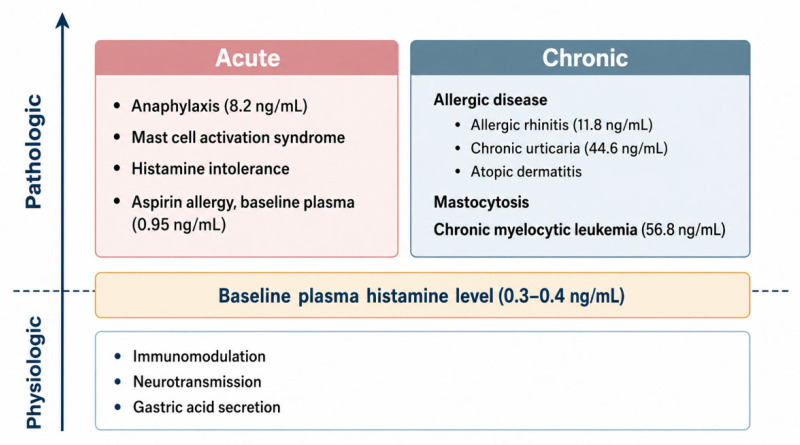
Conditions that may provoke HMS due to elevated histamine levels. Baseline plasma histamine levels can be high in AR [60] and CU [59]. Resting levels are typically lower in drug allergy [56]. AR, allergic rhinitis; CU, chronic urticaria.

In CU, the mean baseline serum histamine level was reported to be (44.6 ± 3.8) ng/mL; then, after four weeks of a histamine-free diet, it decreased to (16.6 ± 1.6) ng/mL (Figure 2) [59]. However, both are very high level as compared to baseline concentrations, as expected.

Plasma histamine levels in AR have been reported to approach those observed in anaphylaxis, as (8.61 ± 3.05) ng/mL in a sneezing group, (4.76 ± 2.54) ng/mL in a nasal congestion group, and (11.82 ± 3.04) ng/mL in a mixed group with both sneezing and nasal congestion [60]. Also, these are much higher than expected.

Mastocytosis is characterized by abnormal mast cell proliferation. Clinical features include flushing, pruritus, abdominal pain, diarrhea, hypotension, syncope, and musculoskeletal pain [61] (Table 2). These manifestations reflect mediator release and/or mast cell infiltration in the skin, gastrointestinal tract, liver, spleen, lymph nodes, and bone marrow. Symptoms and clinical course are heterogeneous, depending on the degree of systemic mediator release and organ dysfunction.

MCAS can involve the skin, gastrointestinal, cardiovascular, respiratory, and neurologic systems, and is commonly classified as primary, secondary, or idiopathic. Diagnostic concepts emphasize episodic symptoms, with mast cell mediators affecting two or more organ systems, including urticaria, angioedema, flushing, nausea, vomiting, diarrhea, abdominal cramping, hypotensive syncope, tachycardia, wheezing, conjunctival injection, pruritus, and nasal stuffiness [62] (Table 2). Histamine intolerance (HIT) is a pathological process that results from a disbalance between the levels of released histamine and the ability of the body to metabolize it. It was reported that accumulated histamine leads to the onset of “histamine-mediated” reactions, which are usually excessive, and decrease quality of life [63]. HIT can cause gastrointestinal symptoms, such as bloating, abdominal discomfort, gas, diarrhea, or constipation [64] (Table 2). Extraintestinal manifestations may include rhinorrhea, rhinitis, nasal congestion, dyspnea, and sneezing. Cutaneous manifestations include pruritus, flushing, urticaria, dermatitis, and swelling. Other systems may be affected, including menstrual cramps, tachycardia, hypotension, collapse, headache, and migraine.

Histamine intoxication refers to excessive histamine accumulation after the ingestion of histamine-rich foods, leading to diverse receptor-mediated symptoms through H1R to H4R [65]. Increased blood histamine can therefore occur even in otherwise healthy individuals after the consumption of foods with high histamine content.

A 120 mg dose of histamine, when instilled into the duodenum, was well tolerated in healthy subjects [66]. In CU, a histamine-free diet reduced baseline serum histamine from (44.6 ± 3.8 to 16.6 ± 1.6) ng/mL after four weeks [59]. Histamine-rich foods include certain fish, such as tuna, mackerel, and Pacific saury; meats, such as pork and chicken; and vegetables, such as spinach. Fermented foods often contain higher histamine levels, including fermented cabbage or radish, soybean paste, red pepper paste, mayonnaise, yogurt, cheese, ketchup, wine, and beer.

Histamine infusion has been used experimentally to provoke dose-related cutaneous flushing, pulsatile headache, tachycardia, and hypotension. In one study, flushing and headache developed at 2.39 ng/mL, increased heart rate at 1.61 ng/mL, and widened pulse pressure at 2.45 ng/mL [49] (Figure 1). These responses occurred in association with plasma histamine levels in the (1.6 to 2.5) ng/mL range.

## 4. IHC Therapy and Clinical Evidence for HMS

**Figure 3:**
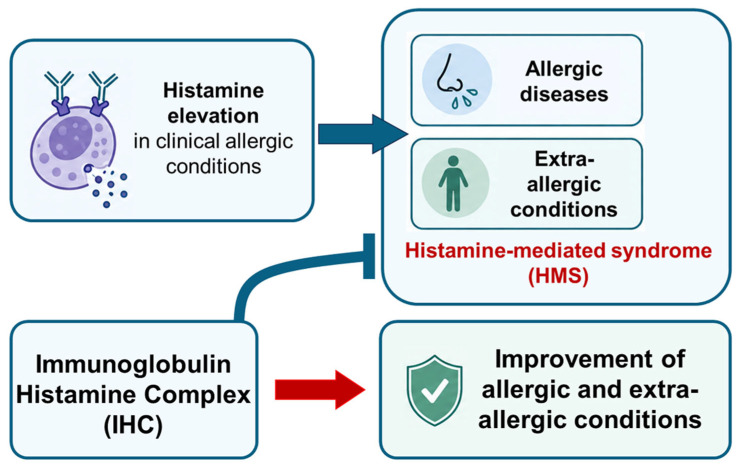
Conceptual framework linking histamine burden to multi-organ manifestations in HMS and the proposed role of IHC. Elevated histamine levels may contribute to both allergic and extra-allergic manifestations through receptor-mediated effects across multiple organs. IHC is proposed to reduce histamine burden, potentially improving histamine-associated symptom clusters in specific contexts, although direct mechanistic evidence in clinical practice is still limited.

IHC is a histamine-fixed immunoglobulin preparation comprising 0.15 μg of histamine dihydrochloride and 12 mg of IgG (Figure 3). Histobulin [67], histaglobulin [68], and histaglobin [6] are generic names for IHC across different countries and reports.

IHC was developed from histamine-fixed serum and can inhibit antigen-induced histamine release from human peripheral blood basophils and rat peritoneal MCs [5]. Histamine conjugated to IgG has been proposed to induce antihistamine antibodies, potentially contributing to reduced responses after allergen challenge [7]. Ayoub et al. reported that histaglobin modulated the Th1/Th2 balance toward a Th1-favoring profile in an ovalbumin allergy mouse model [69]. They also noted the anti-inflammatory effects of histaglobin, which include the inhibition of NF-κB nuclear translocation and the downregulation of pro-inflammatory cytokines [70]. Given histamine’s tendency to promote Th2 skewing in certain contexts, its reduction by IHC may contribute to a Th1-favoring balance [71]. However, direct mechanistic evidence in clinical settings remains limited.

Clinically, IHC has been used as an anti-allergic therapy, with reported efficacy in AR [6], CU [72], bronchial asthma [73], and AD [74]. Recent mechanistic studies on IHC have been limited. As a result, the proposed mechanisms discussed here are primarily based on earlier experimental work, while more recent publications have mainly focused on clinical applications and outcomes.

### Reinterpretation of IHC Outcomes Within the HMS Framework

Published clinical reports have not only given the clinical concepts of HMS but also suggested several insights into the therapeutic potential of IHC (Figure 3). During IHC treatment, not only have multiple allergic diseases improved, but also extra allergic conditions have been reported to be improved in parallel. When these outcomes are analyzed carefully, they provide clinical clues consistent with HMS, which by definition involves manifestations affecting more than one organ system.

Clinical benefit of IHC in AD has been reported [74]; in those cases, AR frequently coexisted, as summarized in Table 3. In another report, multiple allergic diseases improved simultaneously, and anti-inflammatory effects with pain relief were described in Pfeffer–Weber–Christian disease (PWCD), treated with IHC [67]. All cases showed the involvement of multiple organs or tissues.

Histamine contributes to the pathogenesis of CU [66]. Remission induction in CU following IHC therapy has been reported [75], while remission-focused outcomes have been discussed systematically [72].

Depression and anxiety symptoms improved during IHC therapy in CU [76]. Panic disorder concurrent with CU also improved in parallel with CU control in a reported case [77].

In patients with PWCD and multiple allergic diseases treated with IHC, pain relief accompanied the improvement of allergic disease [67]. Similarly, in primary eosinophilic colitis treated with IHC, pain relief accompanied clinical improvement [78].

These observations are consistent with the possibility that histamine signaling contributes to pain pathways [79]. Nociceptive pain developed in CU when the clinical severity was high. However, pain disappeared with the decrease in clinical severity with IHC therapy [80]. Pain seems to be one of the symptoms of HMS.

Collectively, reports of IHC use in non-allergic as well as allergic conditions provide clinical clues consistent with HMS, as summarized in Table 3. The baseline plasma histamine levels reported in AR and CU can be comparable to, and in some reports overlap with, levels reported in anaphylaxis. Accordingly, HMS may be clinically relevant in common conditions such as AR and CU. Namely, HMS seems not to be a special or rare condition, but a frequent situation that clinicians may encounter in general clinics. So, it seems to be necessary to look more carefully for the symptoms and signs of non-allergic symptoms and signs from organs and tissues that have receptors for histamine other than those of AR and CU. Currently, IHC is an effective and safe biologic for the control of histamine levels, rather than using a mixture of each receptor blocker.

## 5. Histamine Control and Therapeutic Approach for HMS

Conventional approaches for managing histamine-associated symptoms include dietary histamine reduction, H1 and H2 antihistamines, and mast cell–directed therapies. In this context, IHC can be viewed as a targeted approach to addressing histamine burden, although the current evidence largely comes from clinical reports and limited mechanistic studies.

Current approaches to histamine control include the elimination of histamine-rich foods and pharmacologic agents that modulate histamine pathways, such as H1R blockers, mast cell stabilizers, and mirtazapine. Supportive measures include vitamin C, flavonoids, diamine oxidase (DAO) supplemented products, and certain probiotics, such as *L. faecalis*-based formulations [25]. Antihistamines targeting H1R and H2R are well established for allergic symptoms [81] and gastric acid-related disorders [10]. In contrast, antagonists for H3R and H4R were present as experimental agents as antagonist for H3R, such as UW–MD71, thioperamide, ciproxifan, ABT–239, A–431404, and SAR110894 [82], and for H4R [1]. No approved H3R antagonists or H4R antagonists are in routine clinical use.

Across published clinical reports, IHC appears to be a potentially effective approach to lowering histamine levels (Figure 3). It is very complex to control histamine-mediated symptoms and signs using a medication mixture for individual H1R to H4R. There has been no concept to control plasma histamine levels prior to the development of IHC. Antihistamines for H1R or H2R are used just to block histamine actions through histamine receptors. In the main, the HR1 blocker is used for allergic diseases, and the H2R blocker for gastric diseases. Plasma histamine level is not controlled by H1R and H2R, nor is it even increased [49].

In 1951, Parrot reported the histamine-fixing capacity of heparin [83]. Thereafter, heparin was replaced by immunoglobulin G as the current form [84]. The histaminopexy effect was reported as an action mechanism of IHC [85]. The induction and production of antihistamine antibody by IHC therapy were reported [70]. Inhibition of mast cell degranulation and histamine release by IHC was also reported [5]. Also, modulation of Th1/Th2 imbalance by IHC was reported [7]. Since IHC was recommended for use in the treatment of allergies with these action mechanisms [86], IHC has been tried in allergic disease, including AR, CU, and AD [68].

Based on the many actions of histamine through four receptors on various cells and tissues, it is time for the clinical manifestations of non-allergic phenomena by histamine to be separated from allergy as HMS or included in all the symptom clusters as those of allergy. Considering the possibility and clinical reports that each individual disease may be present independently, the authors regard it as better for the diseases to be differentiated independently according to their symptoms and signs, to avoid the obscurity of diseases.

## 6. Conclusions

Histamine exerts diverse biological actions through four receptors distributed across tissues and organs. The baseline histamine concentration in common and often non-severe allergic diseases, such as AR and CU, can be unexpectedly high and can be comparable to levels reported in anaphylaxis. Accordingly, HMS may occur more frequently and be closer to routine clinical practice than is generally recognized. HMS should therefore be considered when diagnosing and managing common allergic diseases, particularly AR and CU. IHC may represent a rational therapeutic option for HMS because it targets elevated histamine burden, rather than blocking receptor signaling one receptor at a time.

## Figures and Tables

**Table 1:** Conditions associated with increased plasma histamine levels. AD, atopic dermatitis; CML, chronic myelocytic leukemia; MCAS, mast cell activation syndrome.

Action	Mechanism	Representative Condition
Increased histamine production	Acute allergic mediator release	Anaphylaxis Acute urticaria
	Chronic allergic inflammation	Allergic rhinitis (AR) Chronic urticaria (CU) Atopic dermatitis (AD) Asthma
Increased histamine-producing cells	Cell proliferation or activation	Mastocytosis Chronic myelogenous leukemia (CML) Mast cell activation syndrome (MCAS)
Increased intake of histamine-containing food	Dietary exposure	Histamine intolerance

**Table 2:** Organ involvement and representative symptoms and signs in HMS. GI, gastrointestinal; MCAS, mast cell activation syndrome.

System/Organ		Mastocytosis	MCAS	Histamine Intolerance	Anaphylaxis
**Skin**		Urticaria	Urticaria	Urticaria	Generalized hive
		Angioedema	Angioedema	Swelling	Angioedema
		Pruritus	Pruritus	Pruritus	Pruritus
			Flushing	Flushing	Flushing
**GI**		Abdominal pain	Abdominal cramping	Abdominal discomfort	Abdominal pain
		Diarrhea	Diarrhea	Diarrhea	Diarrhea
			Nausea	Constipation	Nausea
			Vomiting		Vomiting
**Cardiovascular**		Hypotension	Hypotension	Tachycardia	Hypotension
			Tachycardia		Tachycardia
**Respiratory**	**Lung**		Wheezing	Dyspnea	Wheezing
					Dyspnea
	**Nose**		Nasal stuffiness	Congestion	Congestion
				Sneezing	
	**Larynx**				Shortness of breath
**Eye**			Conjunctival injection		
**Neurologic**		Syncope	Hypotensive syncope	Headache	Headache
		Headache	Headache	Migraine	Confusion
**Musculoskeletal**		Pain			
**Reproductive**				Menstrual cramps	Uterine contraction
**Others**					Metallic taste
					Dysphagia

**Table 3:** Reported clinical applications of IHC reinterpreted in the context of HMS. AD, atopic dermatitis; AR, allergic rhinitis; CU, chronic urticaria; FA, food allergy; GI, gastrointestinal; PEC, primary eosinophilic colitis; PWCD, Pfeffer–Weber–Christian disease. Each report includes manifestations involving two or more organ systems consistent with the HMS framework. Collectively, these reports suggest that positioning IHC as a histamine-burden-targeted approach may improve multi-organ manifestations in specific settings, although it is important to note that the evidence primarily relies on clinical reports.

Condition	Organ/System	Diseases/ Symptoms	Main Target Disease for IHC Therapy						
			AD	PWCD	CU	PEC	Depression /Anxiety	Panic Disorder	Food Allergy
**Allergic**	Respiratory	AR	AR	AR	AR	AR	AR	AR	AR
	Skin	CU		CU	CU	CU	CU	CU	CU
	Skin	AD	AD						AD
	GI	FA							FA
**Non-allergic**	Inflammation			Paniculitis		Colitis			
	Pain	Pain		Pain		Pain			
	GI	Abdominal pain;				Abdominal pain			
	GI	Epigastric pain							
	Neurologic/Psychiatric	Depression					Depression	Depression	
	Neurologic/Psychiatric	Anxiety					Anxiety	Anxiety	
	Neurologic/Psychiatric	Panic						Panic	

## Data Availability

No new data were created or analyzed in this study. Data sharing is not applicable to this article.

## Abbreviations

AR	allergic rhinitis
AD	atopic dermatitis
CML	chronic myelocytic leukemia
CU	chronic urticaria
FA	food allergy
HMS	histamine-mediated syndrome
H1R	histamine receptor 1
H2R	histamine receptor 2
H3R	histamine receptor 3
H4R	histamine receptor 4
IHC	immunoglobulin/histamine complex
MC	mast cell
MCAS	mast cell activation syndrome
PWCD	Pfeffer-Weber-Christian disease
